# Suitability of Smartphone Inertial Sensors for Real-Time Biofeedback Applications

**DOI:** 10.3390/s16030301

**Published:** 2016-02-27

**Authors:** Anton Kos, Sašo Tomažič, Anton Umek

**Affiliations:** Faculty of Electrical Engineering, University of Ljubljana, Ljubljana 1000, Slovenia; saso.tomazic@fe.uni-lj.si (S.T.); anton.umek@fe.uni-lj.si (A.U.)

**Keywords:** biofeedback system, smartphone sensors, MEMS sensors, sensor noise, Allan variance, bias error, bias compensation, real-time biofeedback application

## Abstract

This article studies the suitability of smartphones with built-in inertial sensors for biofeedback applications. Biofeedback systems use various sensors to measure body functions and parameters. These sensor data are analyzed, and the results are communicated back to the user, who then tries to act on the feedback signals. Smartphone inertial sensors can be used to capture body movements in biomechanical biofeedback systems. These sensors exhibit various inaccuracies that induce significant angular and positional errors. We studied deterministic and random errors of smartphone accelerometers and gyroscopes, primarily focusing on their biases. Based on extensive measurements, we determined accelerometer and gyroscope noise models and bias variation ranges. Then, we compiled a table of predicted positional and angular errors under various biofeedback system operation conditions. We suggest several bias compensation options that are suitable for various examples of use in real-time biofeedback applications. Measurements within the developed experimental biofeedback application show that under certain conditions, even uncompensated sensors can be used for real-time biofeedback. For general use, especially for more demanding biofeedback applications, sensor biases should be compensated. We are convinced that real-time biofeedback systems based on smartphone inertial sensors are applicable to many similar examples in sports, healthcare, and other areas.

## 1. Introduction

In a biofeedback system, sensors attached to a person measure body functions and parameters (*bio*). These sensors are connected to a processing device to analyze the data. A feedback device is used to communicate the results back to the person (*feedback*) through one of the human senses (*i.e.*, sight, hearing, or touch). The person tries to act on the received information (*biofeedback signal*) to change the body function or parameter in the desired way.

Biofeedback as a discipline based on technology has its roots in the second part of the 20th century. Biofeedback was first described as a process connected to controlling human physiological activities for the purpose of improving health and performance [1,2] and later applied to physical body activity in sports biomechanics [3,4]. Biofeedback can be classified into two main groups: Biomechanical and physiological [5]. In this work, we use the term biofeedback in connection to body activity in the sense of physical movement. According to [5], this is classified as a biomechanical movement biofeedback.

Biomechanical movement biofeedback is useful for motor learning and training in sports, rehabilitation, and other areas [6,7,8,9]. Learning a new movement (motor learning) requires many thousands of repetitions [6] and is usually assisted by a trainer. The role of the trainer is to stimulate the execution of the correct movements and discourage the execution of incorrect movements. Trainer instructions can be given during the execution of the movement (concurrent) or after the movement has been completed (terminal). The trainer can be assisted or, in special cases, even replaced by a biofeedback system. A biofeedback system works in real time if it is capable of giving concurrent feedback to a user during the execution of a movement.

Because numerous correct executions are required to properly learn a certain movement, it is beneficial to detect and possibly prevent improper movement executions. A real-time biofeedback system that gives concurrent feedback can possibly reduce the frequency of improper movement executions and speed up the process of learning the proper movement pattern. Real-time biofeedback is sensible only when human reactions can be performed in-movement (inside the time frame of the movement execution). It is successful if the user, given the biofeedback information, is able to either concurrently correct the improper movement or abandon its execution.

The biofeedback system communicates with the human nervous system through various modalities, *i.e.*, visual, auditory or tactile. User sensing ability and sense occupancy during the performed task condition the choice of modality. In most motor learning tasks, the user's visual channel is preoccupied with the task itself and cannot be efficiently used for biofeedback. Another drawback of the visual modality is its high cognitive load. An alternative is a tactile feedback modality [8,10]. This modality has a relatively demanding implementation, and it can be distracting to the user. Many times the best choice is auditory feedback because this modality is usually unemployed during most exercises [11,12]. It is also relatively easily implementable and has a low cognitive load.

Biomechanical biofeedback systems can be implemented in many different ways. The most common are various video systems with terminal feedback [13,14]. Such systems usually record the exercise or a training episode, which is then replayed and visually analyzed shortly after its execution. More sophisticated are systems with video processing that calculate the information about the desired movement execution parameters [13,14]. Another group is motion tracking systems that use reflective markers to track body movement trajectories. Examples of such systems are Vicon [15] and Qualisys [16]. Such systems may offer real-time functionality, but they are mainly designed to be used in closed and confined spaces with conditions favorable for visual marker tracking. They are also relatively expensive and demand a skilled professional to operate them. An alternative motion tracking technology is based on inertial sensors [17,18,19,20,21]. Some advantages of inertial sensor systems are inexpensiveness, accessibility, portability, and ease of use. The drawbacks include possible high inaccuracies of the results when used without error compensation.

Today’s inertial sensors predominantly fall into the group of *micro electromechanical systems* (MEMS). They are small and inexpensive; however, their performances are relatively poor compared to professional navigational sensors [11]. The precisions of MEMS gyroscopes and accelerometers are primarily affected by their biases, which induce errors in the derived angular and spatial positions.

Presently, thanks to smartphones, MEMS inertial sensors are readily available and widespread. According to [22], in many countries, the penetration of smartphones exceeded 50% in 2013, and according to [23], more than 2 billion people will own smartphones by the year 2016. All new smartphones are built with numerous sensors, practically always including accelerometers and gyroscopes. Because of the above factors, building biofeedback systems using the inertial sensors that are integrated into smartphones is desirable. This approach has many advantages. Inertial sensors are already built into smartphones, representing a mobile wearable system with a powerful processing unit (CPU), a large battery, a high definition screen, many input/output interfaces, various choices of wireless connectivity, *etc.* Another advantage is the presumed synchronization of all sensor signals taken from the same smartphone. Less demanding biofeedback applications can be implemented entirely in the smartphone. Use of smartphones could also have some disadvantages. The most notable are their size and weight. When used as motion tracking sensors, smartphones: (a) cannot be physically attached to certain parts and (b) could interfere with the movement being executed due to weight and size. Another possible disadvantage is the imprecision of smartphone sensors, which could be the limiting factor for their use in many biofeedback systems.

To investigate the limitations of smartphone inertial sensors for use in biofeedback systems, the general properties and demands of biomechanical biofeedback applications should be defined. In biomechanical biofeedback applications, inertial sensors are used to detect and possibly track body movements. Various parameters can be used for the evaluation of inertial sensors for particular biomechanical biofeedback applications:

(a) *Movement dynamics* describes the swiftness of change in a movement, e.g., fast movements in sports and slow movements in rehabilitation biofeedback systems.
(b) In connection to (a), the required biofeedback system *sampling frequency* varies from a few tens to a few hundred Hertz.
(c) *Measurement range* defines the boundary levels of the measured sensor signal (*i.e.*, acceleration and angular velocity).
(d) The duration of movement execution defines the width of the *analysis time frame T_w_* that can vary from less than a second to a few minutes or even hours.
(e) The required ***accuracy*** of the measured or calculated movement parameters includes
  - The *position* accuracy calculated from accelerometer readings and, in the case of sensor fusion, from gyroscope readings; the maximal accumulated error ranges from a few millimeters to a few centimeters.
  - The *posture angle* accuracy calculated from accelerometer readings; the maximal accumulated error ranges from less than one degree to a few degrees.
  - The *rotation angle* accuracy calculated from gyroscope readings; the maximal accumulated error ranges from less than one degree to a few degrees.
(f) Measurement *precision i*s more important in biofeedback applications than accuracy itself. When consistently repeating the same movement, measured values must be precise, even if they are not accurate.

One example of a biofeedback application in sports that can illustrate the application of the above defined biofeedback application parameters is a golf swing. It is a static exercise, but the body movements can be rather fast (high dynamic). Consequently, the sampling frequency must be high enough. Depending on the sensor placement, the accelerometer must cover different measurement ranges. For example, when attached to the lower part of the golf club, accelerations are much higher than when attached to the wrist or the arm of the player. The analysis time frame is between 1.5 and 3 s, depending on the player. The required accuracies are between 2 to 3 degrees for posture and angular rotation. The required measurement consistency is in the range of 2 degrees [24]. We developed an experimental real-time biomechanical biofeedback application with feedback for golf swing training that uses smartphone sensors.

### 1.1. Related Research

Various applications of smartphone sensor usage in biofeedback systems can be found in healthcare, rehabilitation and sports. For example, in healthcare, accelerometers in smartphones are used to detect and communicate the falling events of patients [18]. In rehabilitation, accelerometers in smartphones are used to improve a person’s balance [7], for gait training of persons with Parkinson’s disease [19], for terminal exercise performance assessment [20], *etc*. In sports, the movement dynamics are usually greater than in healthcare and rehabilitation, possibly limiting the use of devices with MEMS inertial sensors for biomechanical movement biofeedback. Accelerometers are used to drive real-time visual feedback aimed at reducing tibial stress in runners [9] and to detect and evaluate the performances of dance students [21]. Paper [13] reviews the use of sensors for motor learning and analyzes 82 sensor-based prototypes, exploring their learning support. A recent review of the ability of microsensors to detect sport-specific movements is presented in [14]. Commercially available microsensors are capable of quantifying sporting demands that other monitoring technologies may not detect.

Extensive research has been conducted on the performances of standalone MEMS [25,26,27,28,29,30,31,32,33,34,35]. There has been much less research specifically on smartphone embedded MEMS. Measurements of standalone MEMS are generally done in controlled conditions, *i.e.*, stable temperature, use of mechanical devices for accurate MEMS positioning, and at standstill (environment without vibration). Smartphone embedded MEMS rarely experience such controlled conditions. Therefore, it is essential that their performances are evaluated in normal operating conditions, *i.e.*, with temperature changes due to the heating of other smartphone circuitry, measurements in slightly inaccurate positions, and in the presence of vibrations. Limited research about the latter issues can be found in [36].

### 1.2. Problem Formulation and Research Contributions

Biomechanical biofeedback applications with real-time motion tracking and feedback require sensor signals with certain accuracies and precisions. Raw sensor signals from MEMS sensors integrated in smartphones are processed by the smartphone’s operating system (OS) and made available to developers and applications as a *smartphone sensor*. In this work, the term *sensor* denotes the smartphone sensor defined above and the term *MEMS sensor* denotes the physical sensor. Similarly, the term *sensor signal* denotes a signal from the smartphone sensor.

While the use of *smartphone sensor* is beneficial from the application programming point of view (standard interface), it is less convenient from the application functional point of view (exact sensor properties are unknown). Complex and demanding smartphone applications, such as real-time motion tracking with biofeedback, require information about the exact sensor parameters. For the abovementioned reasons, smartphone sensor parameters cannot be taken directly from the manufacturer’s specifications of the MEMS sensor embedded into a smartphone model but should be measured for each model separately.

MEMS sensor parameters and the depending smartphone sensor parameters are not the only limiting factors for more demanding smartphone applications. Applications access MEMS sensor data and signals through the smartphone OS and APIs. One group of limitations is linked to a limited choice of available set of MEMS sensor parameters. For example, MEMS accelerometer may have a set of different measurement ranges: ±2 g, ±4 g, and ±8 g, but the operating system only allows the use of ±2 g. Another group of limitations is linked to the usage of smartphone resources, particularly its processing power and battery usage. For example, the smartphone OS does not allow too frequent access to sensor data, therefore the smartphone sensor data rate is limited to levels below the MEMS sensor data rates. Therefore the effective smartphone sensor sampling frequency can be much lower that the sampling frequency offered by the MEMS sensor. It has been reported that the effective sampling frequencies of smartphone sensors are up to approximately 100 Hz [37]. APIs, software development tools, and libraries for smartphone applications can also be a problem. They may not include all the necessary routines, protocols, and tools for building an effective application using smartphone sensors. Other limitations may exist, but they are not the focus of this work. The principal goal of the present research is to develop a methodology for the evaluation of the adequacy of smartphone inertial sensor parameters for use in real-time biofeedback applications. Our interests are primarily in the empirical evaluation of the usability of a smartphone as a system. For that purpose, we use the same measurement procedures as those used in measurements of the parameters of standalone sensors in laboratory environments. However, these procedures need to be tailored to the real-time biomechanical biofeedback applications of smartphones used outside the laboratory environment. The main contributions of the present research are:

(a) measurement and modeling of the inertial sensor signals that are available within the smartphone operating system,
(b) estimation and rating of the most influential smartphone sensor error sources,
(c) definition of various scenarios for smartphone sensor bias compensation,
(d) definition of real-time biofeedback application feasibility based on predicted smartphone sensor errors in different bias compensation scenarios,
(e) development of the experimental biofeedback application.

The rest of the paper is organized as follows: Section 2 presents the experimental design and components of the general real-time biofeedback system. Section 3 provides details about the smartphone inertial sensor measurements with emphasis on the sensor biases and the errors caused by them. A discussion of the measurement results and the proposed bias compensation scenarios is given in Section 4. Section 5 presents the developed experimental biofeedback application, followed by its test results in Section 6. Conclusions and future work are presented in Section 7.

## 2. Experimental Design of the Real-Time Biofeedback System

**Figure 1:**
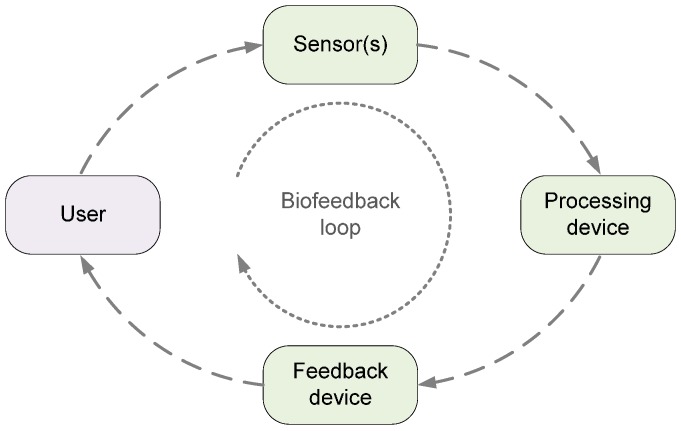
Operation of the biofeedback system. The user starts to execute a movement. One or more sensors attached to the user feed their signals to the processing device for real-time signal analysis. The analysis results drive the feedback device activity. The user may react to the feedback signals (audio, video, or tactile), ideally by correction of the unwanted action, but most likely by interrupting the action and correcting it in the next try. User (re)action alters the sensor signals, thus closing the feedback loop of the system.

The biofeedback system, as defined in the introduction, requires one or more inertial sensors, a processing device, one or more feedback devices, and when implemented as a distributed system, one or more communication channels. The architecture of the experimental biofeedback system, used by the application in Section 5, is shown in Figure 1. It is composed of the following elements:

(a) *Inertial sensors* attached to the user’s body. They can be integrated into a dedicated sensor device or, as in our case, into a smartphone. In the experiments, we use an iPhone 4 with an integrated 3-axis ST Microelectronics LIS331DLH accelerometer [28,29] and a 3-axis ST Microelectronics L3G4200D gyroscope [30,31]. Their main parameters are listed in Table 1. However, according to the iPhone 4 specifications, its smartphone sensor parameter values are limited to ±2 g for accelerometer, ±2000 deg/s for gyroscope, and to the sampling frequency of approximately 100 Hz.
(b) The standalone *processing device* is a laptop running Microsoft Windows 7 operating system and National Instruments LabVIEW™ 2014 measurement and analysis software.
(c) Wireless headphones are used as the *feedback device*.
(d) The above elements are interconnected over the following *wireless channels*:
  - The smartphone with sensors and the laptop communicate over the IEEE 802.11 wireless local area network. We use the iOS application Sensor Monitor (Pro) 1.0.9 to send the smartphone sensor signals to the laptop.
  - The laptop and the headphones communicate over the proprietary radio channel protocol.

For the evaluation of the smartphone inertial sensor performance, it is crucial that measurements are done on the same equipment and the same system architecture. Therefore, the measurements of the smartphone inertial sensor performance parameters were carried out on the elements of the above described biofeedback systems with the following differences: (a) smartphone was not attached to the user but used in a way that is appropriate to the specific measurement; (b) biofeedback loop operation algorithms in the processing device were replaced by algorithms for the measurement of smartphone inertial sensor performance; and (c) a feedback device was not used.

The operation of the biofeedback loop largely depends on the inertial sensor quality and performance parameters of the smartphone. Precise sensor readings are critical for accurate analysis and correct feedback signals. Therefore, in the next section, we focus on the performances, precisions, parameter measurement, bias determination and compensation of smartphone inertial sensors. We aim to determine the degree of usability of smartphone inertial sensors for real-time biofeedback applications.

## 3. Measurement of Smartphone Inertial Sensor Performance Parameters

Inertial sensor errors have already been described well in various studies [38,39] and can be classified into two groups: deterministic and random. Deterministic errors are bias, scale factor errors and axes misalignment errors. Random sensor errors are generated by various type of noise and depend on the sensor technology [33]. There are other documented sources of MEMS errors [32,35] such as the effects of gravitational forces and vibrations. Deterministic errors can be greatly reduced by sensor calibration procedures, while noise reduction is limited and requires adequate noise filtering techniques. Noise reduction is also possible by using sensor arrays [40]. The most relevant deterministic error source for low-cost MEMS gyroscopes and accelerometers are their biases [38].

### 3.1. Accelerometer and Gyroscope Bias Measurements

**Figure 2:**
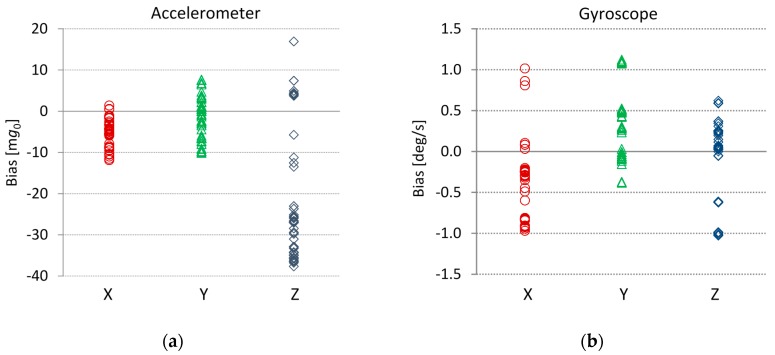
3D gyroscope and 3D accelerometer biases obtained from multiple measurements on several smartphones. Values are shown in a scatter diagram with each of the axes plotted in a separate vertical column. Biases are calculated by averaging *N* = 600 sensor signal samples at a sampling frequency *f_s_* = 60 Hz; the corresponding averaging time is therefore τ = 10 s. (**a**) Accelerometer biases presented in m*g*_0_ have slightly different dynamic ranges; (**b**) Gyroscope biases presented in deg/s have similar dynamic ranges.

The *bias measurement system* includes an iPhone 4 smartphone with integrated accelerometer and gyroscope, the *Sensor Monitor* application that collects and sends sensor data from the iPhone, and a LabVIEW™ application. Bias measurements were carried out using the LabVIEW™ application running on a laptop, which received sensor data from the smartphone over the local wireless network. We also constructed a special casing that allowed us to simply orientate the smartphone in any of the principal positions. The measurements were performed at room temperature (approximately 22 °C) on a flat horizontal surface in six positions to eliminate the influence of gravity. Bias measurement results from six iPhone 4 smartphones are shown in Figure 2. Measurements for all accelerometer and gyroscope axes were averaged over *N* = 600 samples in the time interval τ = 10 s.

The gyroscope biases are within the range of Δ*G*_0_ = ±1.15 deg/s. The accelerometer biases are within the range Δ*A*_0_ = ±12 m*g*_0_ for the X- and Y-axes, and Δ*A*_0_ = ±40 m*g*_0_ for the Z-axis. The aim of these results is to present bias variations on different smartphone devices of the same type (iPhone 4).

### 3.2. Accelerometer and Gyroscope Constant Bias Errors

The first evaluation of the measured biases in the scope of movement tracking and detection in biofeedback systems is done based on position and angle deviation from their real values. The largest measured accelerometer bias in Figure 2 is 38 m*g*_0_. The position drift Δ*s* is a time quadratic function and consequently very sensitive to accelerometer constant bias Δ*a:*

(1) $$\Delta s(t)=\frac{1}{2}\Delta a\cdot t^{2}$$

The maximal position error from the measured accelerometer biases is 19 cm after 1 s. Therefore, accelerometer bias compensation is practically mandatory for movement tracking. The accelerometer signal can also be used to measure the direction of the gravity vector $\vec{g_{0}}$. Its direction error δ is proportional to the accelerometer bias vector $\Delta \vec{a}$*:*

(2) $$\delta =\text{acos}\left(\frac{\vec{g_{0}}\cdot (\vec{g_{0}}+\Delta \vec{a})}{\left|\vec{g_{0}}|\cdot |\vec{g_{0}}+\Delta \vec{a}\right|}\right)$$

The predicted gravity vector direction error from the accelerometer measurement result in Figure 2 is therefore less than 2.2 degrees.

The constant gyroscope bias induces a linear angular drift ΔΦ*:*

(3) Δφ(t)=Δω⋅t

The maximal measured linear angular drift for several different iPhone 4 smartphones is approximately 1 deg/s. The gyroscope angle error is not too high. Gyroscopes are potentially usable for short time movement analysis applications that do not require extreme precisions.

### 3.3. Bias Variations

MEMS accelerometer and gyroscope biases vary with time [32]. One of the most well-known and influential factors in bias instability is temperature. Both sensor biases and scale factors are temperature sensitive parameters. For that reason, various MEMS techniques are used to compensate sensor temperature dependency [41,42]. The thermal bias stability is also limited by temperature hysteresis error, which cannot be compensated [43]. Low-frequency bias changes are frequent due to partially uncompensated MEMS temperature variations.

Precise measurements of standalone sensor ICs (integrated circuits) can be done in temperature chambers with temperature regulation. Sensors in smartphones also experience changes in temperature because of other pieces of hardware integrated into the enclosed casing of the smartphone. At room temperature, the heating of the smartphone is predominantly caused by power dissipation from the CPU, screen, RF circuitry, GPS module, and other integrated circuits. Overheating of the batteries at charge time is also a possible cause. In its operating state, when applications are running and consuming power, the surrounding ambient air temperature has a smaller effect on the sensor temperature. Large internal temperature fluctuations are accompanied by large temperature-induced bias drifts in accelerometers and gyroscopes.

**Figure 3:**
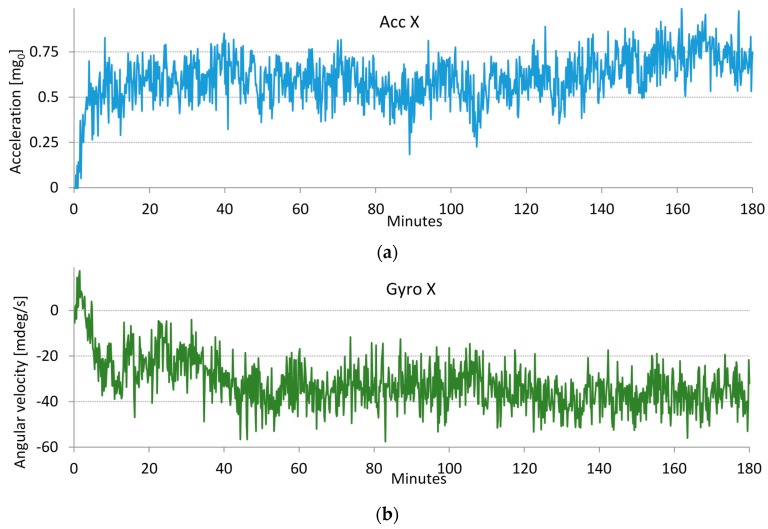
Measured bias values in the 3-hour temperature stress test. The bias values are found by averaging 600 sensor signal samples in 10 s intervals. Temperature changes induce noticeable bias drifts in the accelerometers and gyroscopes. The graphs show the bias drifts of the (**a**) accelerometer X-axis and (**b**) gyroscope X-axis.

During the bias measurements in Figure 2, we noticed differences in the biases of the same smartphone and between different smartphones. While differences between devices are due to variations in the physical properties of the embedded MEMS [28,29,30,31,32], the differences in successive measurements of the same device are caused by various inertial sensor instabilities, most likely because of slightly different internal phone temperatures between measurements. To test our assumptions, we conducted a simple temperature stress test. First, we cooled the switched-off smartphone to 8 °C. After turning the phone on and placing it in a location at room temperature (21 °C), we measured the biases for three hours. During the tests, the smartphones were leveled using a leveling scale with an accuracy of 1 mm per meter or 0.057 degrees. We performed measurements in different smartphone orientations with one of the axes parallel to the Earth's gravity vector_._ Examples of accelerometer and gyroscope bias drifts are shown in Figure 3. The bias drifts in Figure 3 exhibit the strongest temperature dependences in the first half hour of the test. In this period, the temperature changes induce bias drifts that can be larger than the bias variations caused by other sources of sensor error, including noise. The results of the stress test measurements of several smartphones show that bias drifts with similar dynamics are found on the other axes.

Temperature stress tests show that, as anticipated, the changes in temperature cause large bias variations. Stable temperature conditions are reached after approximately one hour. Biases measured after the transition period do not change considerably. As shown in Figure 3a, the variations in the accelerometer X-axis bias due to sensor noise do not exceed 0.4 m*g*_0,_ and bias drift after one hour reaches 0.7 m*g*_0_. The variations in the gyroscope X-axis bias are shown in Figure 3b. The variations in the gyroscope bias due to sensor noise do not exceed 30 mdeg/s, and the gyroscope bias drift after one hour is approximately 40 mdeg/s.

Bias error measurements in stable ambient temperature conditions were carried out on six smartphones. The variations in the accelerometer bias due to noise are in the range of 0.4 to 0.5 m*g*_0_. The variations in the gyroscope bias due to noise are in the range of 30 to 60 mdeg/s. Larger differences are found in the bias drifts. Measured accelerometer drifts do not exceed 4 m*g*_0_ per hour, gyroscope drifts remain below 86 mdeg**/s** per hour.

### 3.4. Allan Variance

For detailed analysis of the smartphone inertial sensor errors, we used Allan variance measurements. Biases are measured by averaging a finite sequence of samples when a device is in a standstill position. Bias variations are caused by various random processes in the operation of the sensor. Bias approximations can be calculated by averaging the sensor signal samples:

(4) $$y\left[m\right]=\frac{1}{N}\sum_{n=0}^{N-1}x\left[n+m\cdot N\right]$$

Allan variance σ_A_^2^(N) is a measure of the variations of the mean values *y*[*m*] of consecutive blocks of *N* signal samples *x*[*n*] [33,44,45]:

(5) $$\sigma_{A}^{2}\left[N\right]=\frac{1}{2}\bar{{\left(y\left[m\right]-y\left[m-1\right]\right)}^{2}}$$

The variance is approximated from a finite number of mean values *y*[*m*]:

(6) $$\sigma_{A}^{2}\left[N\right]\approx \frac{1}{2\cdot (M-1)}\sum_{m=1}^{M-1}{\left(y\left[m\right]-y\left[m-1\right]\right)}^{2}$$

The approximation error of Equation (6) is estimated to be [33]:

(7) $$\delta_{\sigma }=\frac{1}{\sqrt{2(M-1)}}$$

Depending on the nature of the random process, the bias noise has a different power spectrum shape. In fact, many different uncorrelated random processes participate at the same time. The Allan variance method helps us to determine the characteristics of the underlying random processes and noise models. The basic bias error models are quantization noise, white noise, bias instability, rate random walk and drift rate ramp. Different noises have different spectrum power density profiles *S*_x_(*f*) and appear in Allan variance plots with different slopes [33,45]:

1. Quantization noise has an accented high frequency power density profile *S_x_*(*f*) *= N_Q_ f* ^2^ and a quadratically decaying Allan variance σ*_A_*^2^(τ) = 3*N*_Q_/τ^2^.
2. White noise, *S_x_*(*f*) *= N*_0_, has a linearly decaying variance σ*_A_*^2^(τ) = *N*_0_/τ. Such noise causes *angle random walk* (ARW) in gyroscopes and *velocity random walk* (VRW) in accelerometers.
3. Low-frequency noise has a power density function *S_x_ = N*_B_/*f* with a constant variance at long averaging time σ*_A_*^2^(τ) = 0.66 *N_B_*. It defines the minimum sensor *bias instability* (BI).
4. *Random rate walk* (RRW) noise has a very long correlation time and, consequently, a narrower frequency power spectrum shape *S_x_*(*f*) *= N*_RRW_/*f*^2^. Therefore, the variance is linearly proportional to integration time σ*_A_*^2^(τ) = 1/3 *N*_RRW_ τ.
5. Some *deterministic errors* also causes slow monotonic bias changes, modeled as *drift rate ramp* (DRR) *x*(*t*) *= Rt*. The Allan variance function for this type of error is a quadratic function of averaging time σ*_A_*^2^(τ) = 0.5 *R*τ^2^. For example, such bias error is caused by temperature changes, and if not compensated, it can become a dominant error source in long time interval.

Various researchers have shown that, in most cases, different noises appear in different regions of τ. In such situations, it is possible to identify the model of the underlying random process from the Allan deviation σ*_A_*(τ)log-log plot:

- High frequency variations caused by the quantization noise and white noise can be determined from the slope of the first segment of the Allan variance plot.
- Low frequency noise models start to dominate after filtering out the high frequency components by widening the averaging time τ.

**Figure 4:**
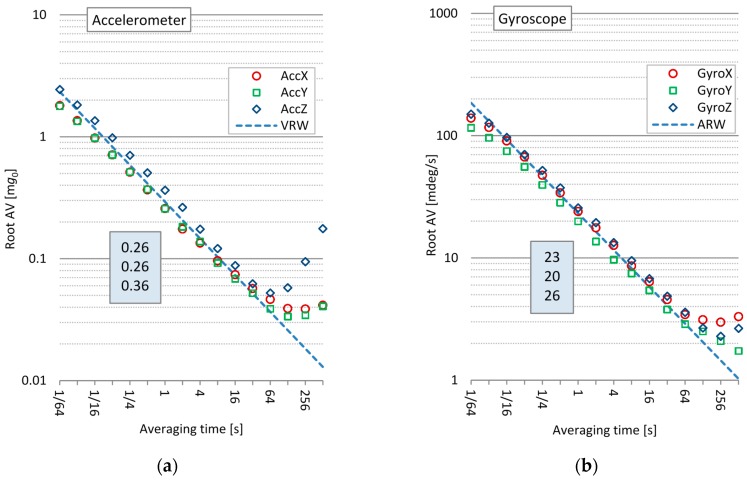
Allan variance measurements for all three axes of the accelerometer and gyroscope of a single smartphone as a function of averaging time in seconds. The dotted line represents the white noise model: (**a**) Accelerometer results conform to the VRW model at short averaging times; (**b**) Gyroscope results conform to the ARW model at short averaging times. Values in rectangles represent Allan deviation at averaging time of 1 s.

Measurements were conducted under stable operation conditions when the smartphone was at standstill, *i.e.*, absence of vibrations, constant room temperature, and constant low power dissipation. Information about the internal smartphone temperature or inertial sensor chip temperature was not available. Measurements of Allan variance were carried out with the time resolution of one sample per decade from τ = 0.1 s to τ = 1000 s. For statistically valuable results, at least *M* = 10 measurements were required at the longest averaging time τ. Therefore, the measurement takes the time *T*_0_ = 10,000 s. To determine all relevant noise terms, a finer resolution of the log(τ) axis and longer measurement times are required. However, the results are still accurate enough to identify the most relevant types of bias errors: white noise and bias drift. Allan variance measurements of the 3D accelerometer and 3D gyroscope for a single smartphone are shown in Figure 4.

As shown in Figure 4a, the Allan deviation of the accelerometer σ_A_(τ) follows the slope of the bias *white noise model* for short averaging times τ ≤ 10 s. The accelerometer *velocity random walk* constant (VRW) can be determined from the Allan deviation plot at τ = 1 s: VRW = $\sqrt{N_{0}}$ = σ*_A_* (τ = 1 s)*.* Model parameters for all three axes are given inside the shaded rectangle in Figure 4a.

As shown in Figure 4b, the Allan deviation of the gyroscope σ_A_(τ) follows the slope of the bias *white noise model* for short averaging times τ < 100 s. The gyroscope *angle random walk* constant (ARW) can be determined from the Allan deviation plot at τ = 1 s: ARW=$\sqrt{N_{0}}$ = σ*_A_* (τ = 1 s)*.* Model parameters for all three axes are given inside the shaded rectangle in Figure 4b.

At longer averaging times, where the averaging filter decreases the power of the high frequency white noise, slow bias fluctuations with low frequency spectra become the dominant error sources for the accelerometers and gyroscopes.

The accuracy of the measured variances varies as estimated by Equation (7). The Allan variance measurements of the most right side points, at *M* = 10 or τ = 1000 s, are less accurate; the accuracy is estimated to be δ_σ_ = 23.6% Equation (7).

The same measurements were performed on six different smartphones (iPhone 4). We have noticed only minor differences in the white noise model parameters VRV and ARW. The differences in the bias instability were more noticeable, which we believe to be primarily the effect of the different temperature sensitivities of the smartphone embedded MEMS. The calculated average sensor white noise model parameters are VRW = 0.25 m*g*_0_/$\sqrt{\text{Hz}}$ and ARW = 28 mdeg/s/$\sqrt{\text{Hz}}$. According to [28] iPhone 4 embedded MEMS accelerometer is ST Microelectronics LIS331DLH MEMS and according to [30] iPhone 4 embedded MEMS gyroscope is ST Microelectronics L3G4200D. Their average noise model parameters are found in Table 1: VRW_MEMS_ = 0.218 m*g*_0_/$\sqrt{\text{Hz}}$ and ARW_MEMS_ = 30 mdeg/s/$\sqrt{\text{Hz}}$. It can be observed that noise parameters of the smartphone sensors offered by the operating system does not differ much from the same parameters of MEMS sensors.

#### 3.4.1. Bias Measurement Error

Based on the measured sensor noise model, we can also determine the averaging times for the bias measurements that are used as the offset values for sensor bias compensation. Bias estimation error is a result of sensor noise passing through an averaging filter. The bias variance decays linearly with integration time only if white noise is a dominant source of error. Unfortunately, averaging cannot eliminate low frequency noise. The results from Figure 4 help us analyze the trade-off between the signal averaging time and the bias measurement accuracy. Reasonable averaging times for bias measurements are between 10 and 100 s. The Allan Variance Equation (6) can be used as an approximation of the standard bias variance [46,47,48]. The predicted bias measurement errors for the accelerometers and gyroscopes with defined confidence interval *k(P)* are Equations (8) and (9):

(8) $$\Delta a=k(P)\cdot \frac{VRW}{\sqrt{T_{avg}}}$$

(9) $$\Delta \omega =k(P)\cdot \frac{ARW}{\sqrt{T_{avg}}}$$

The bias measurement errors are constant after the compensation and induce drifts in Position (1), Orientation (2) and Rotation (3). The bias variations due to the sensor noise shown in Figure 3 are in accordance with the measured sensor noise density described by the ARW and VRW models, where peak-to-peak values are within ±3σ_A_(*T_avg_*) Equations (8) and (9). Under the assumption that the sensor white noise is Gaussian [49,50], *P(k =* 3*)* = 99.7% is the confidence interval.

#### 3.4.2. Influence of the Sensor White Noise on the Derived Parameters

The Allan measurement results confirm that white noise is the dominant sensor error source for short integration times. Gyroscope white noise creates an angle random walk, where the standard deviation grows with the square root of the integration time [51]:

(10) $$\sigma_{\varphi }(t)=ARW\cdot \sqrt{t}$$

Position random variations are the result of accelerometer signal double integration. Under the white noise accelerometer random model, the position variance, as a result of a second-order random walk, grows proportionally with $t^{\frac{3}{2}}$ [51]:

(11) $$\sigma_{\varphi }(t)=\frac{VRW}{\sqrt{3}}\cdot t^{\frac{3}{2}}$$

We assume that the white noise in the accelerometer and gyroscope is Gaussian [49,50], and therefore, both Random Variations (10) and (11) represent 68% confidence intervals.

## 4. Discussion of the Results

**Figure 5:**
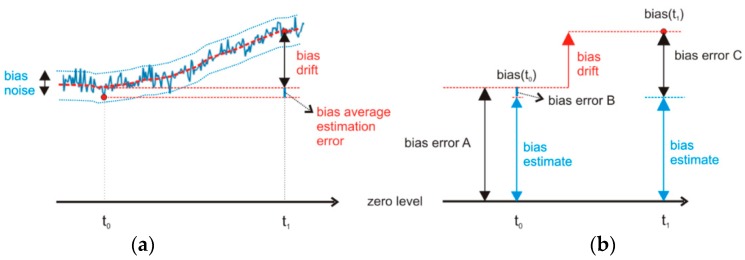
Bias variations and their effects. (**a**) The bias changes with time (blue line) in the short term primarily because of bias noise (ARW) and in the long term because of other influences (red dashed line). Bias drift is the change in bias value between times *t*_0_ and *t*_1_; (**b**) Without compensation, we experience *bias error A*. With the compensation at time *t*_0_, we decrease the bias error for the measured bias estimate to get *bias error B*. By time *t*_1_, the bias drift causes the error to grow to the value of *bias error C*.

The basis for the discussion of the measurement results is Figure 5, which illustrates various error components of the bias.

The measurement results presented in Table 2 (A) show uncompensated bias values from Figure 2 that induce bias error A in Figure 5.

From Table 2, we see that the largest gyroscope biases are approximately 1 deg/s, and the largest accelerometer biases are approximately 40 m*g*_0_. For the *uncompensated gyroscope,* in a short time analysis, for example, in a 3 s analysis interval, the bias induces an angular error of 3.45 degrees.The precisions of the accelerometer and the gyroscope are considerably better after the bias compensation at time *t*_0_ as shown in Figure 5. The compensated bias values at time *t*_0_ induce bias errorB in Figure 5b. The bias measurement error depends on the averaging time in Equations (8) and (9). The predicted bias measurement errors B from Table 2 represent 99.7% confidence intervals under the assumption that the sensor noise is Gaussian. For example, shortly after bias compensation, the expected linear angular drift after a 3 s analysis time does not exceed 0.08 degree, and the expected positional drift remains under 1.0 cm.

If we perform the same analysis at time *t*_1,_ the expected angular drift would be higher because of the bias drift, which would result in bias error C, as shown in Figure 5b. According to the results of the bias drift measurements from Section 3.3, the bias drifts are 4 mg_0_ after one hour for the accelerometer and 86 mdeg/s after one hour for the gyroscope. For example, one hour after compensation, the bias drift induces positional error that does not exceed 17.7 cm and angular error that does not exceed 0.26 degrees in a 3 s analysis interval.

Table 2 shows that the predicted errors one hour after compensation (C) are more than ten times smaller than those before initial compensation (A). If a new bias error induces angular and positional drifts that are no longer acceptable for the application, the bias drift should be corrected with another bias compensation at time *t*_1_. Based on the bias errors in Table 2 and the boundaries set for the exemplary biofeedback application presented in the introduction section, we can write the following findings:

- Relatively high accelerometer biases restrict the use of uncompensated accelerometers for position tracking. Shortly after bias compensation, the accelerometer offers sufficient accuracy for applications with short analysis times. Bias drift reduces the allowed analysis time to below 1 s.
- When an accelerometer is used for tilt or inclination sensing (static angles) in biofeedback applications, bias-induced errors are generally not the limiting factor. Static angle errors due to accelerometer noise and drift are practically negligible. For most biofeedback applications, even the uncompensated accelerometer signals are acceptable.
- Gyroscopes with compensated biases, even some time after compensation, are accurate enough to meet the demands of all biofeedback applications covered in Table 2. Some applications with short analysis times (less than 3 s) can use uncompensated smartphone gyroscopes.

Even with sensor bias compensation, sensor noise sets the lower bound of sensor precision. Table 3 shows the predicted random walk errors of position and angle calculated by Equations (10) and (11). Values are given within a 68% confidence interval under assumption that sensor noise is Gaussian. For the majority of biofeedback applications, the random errors in Table 3, which are caused by noise, are negligibly small compared to the deterministic errors presented in Table 2 (A) and (C). The random walk position and angular errors calculated from the standard deviations in Table 3 can be higher than the linear drift errors induced by the bias measurement presented in Table 2 (B) if the analysis integration time frame is shorter than the bias measurement time *t* < T_avg_.

### 4.1. Bias Compensation Options

Inertial sensor bias variations in the form of noise and drift (see Figure 5) could be the limiting factor for their usability in different types of applications. In biofeedback applications, where we generally use inertial sensors to measure movement patterns, large biases are a limiting factor.

The precision of the sensor readings can be improved to a certain extent by bias compensation, but we have to bear in mind that bias errors can never be fully eliminated as shown in Figure 5. With regard to each individual application and its sensor precision demands, we must choose the right strategy for bias compensation. There are several strategies available for bias compensation:

- *One-time bias compensation* has a time-limited effect. Therefore, it is suitable only for applications that operate in stable environments. For instance, the smartphone is always used inside the same temperature range and at approximately the same processing load.
- *Periodic bias compensation* can be performed at regular time intervals or on an as-needed basis. For instance, bias compensation is needed after every significant change in the inertial sensor temperature.

What are the available application scenarios according to the above options, according to the demands of the application, and according to the required times needed to perform the compensation? What about biofeedback applications that track and detect movements? To achieve different levels of movement detection accuracy, the following application scenarios are possible:

I. The application uses*uncompensated sensor data*. In this case, the bias error corresponds to the bias error A in Figure 5. This compensation scenario could be applicable to short time movements, up to a few seconds long, if the application does not use accelerometers for position calculation and does not demand high angular precision. See values for bias error A in Table 2.
II. Before each session, the application performs a *one-time bias compensation* of the accelerometer and the gyroscope. Shortly after the compensation, the bias error corresponds to the bias error B in Figure 5. With time, the bias error increases and corresponds to the bias error C in Figure 5. The effect of one-time compensation is satisfactory up to one hour if the operating conditions do not differ much from the conditions at which the compensation was performed. In such cases, biases change in a limited value range, see values for bias error C in Table 2. Bias errors one hour after bias compensation are still for an order of magnitude lower than bias errors of uncompensated sensor. One-time bias compensation scenario is applicable to short time movements, up to a few seconds long, even for applications demanding high precision or for medium time movements, up to a few tens of seconds long for less demanding applications.
III. The application constantly compensates biases. At every detected opportunity, the biases are compensated. The measurement times required for this compensation scenario could be between 10 s and 100 s, as proved in Section 3.4. After a longer time without compensation, the application may notify the user that the accuracy of the application operation might be compromised and that a new bias compensation is required. During the application use, gyroscope bias compensation is possible without too much trouble, while accelerometer bias compensation generally requires temporary interruption of application use [52]. The goal in this scenario is to stay as close as possible to the accuracy level of bias error B in Table 2.

## 5. Experimental Biofeedback Application

As an example of a real-time biofeedback system based on smartphone inertial sensors, we designed an application that is designed to help users correct specific golf swing errors. The basic application idea is to monitor the player’s head movements during a golf swing by employing the smartphone’s inertial sensors. Head movements are very often the indicator of incorrect golf swing execution; therefore, the application is developed to detect excessive incorrect head movements and communicate them to the player in real time using audio feedback. The hypothesis is supported by observing the world’s best golfers and their advice [53,54]. The majority of them keep their heads practically still until just after impact.

### 5.1. Application Demands and Constraints

In the context of this work, the most important application constraints are bound to the potential inaccuracies of the accelerometer and gyroscope signals from the smartphone. The inertial sensor signal analysis time interval is defined as the golf swing duration. It takes less than 3 s to perform the golf swing from the *takeaway* (beginning of the swing) to the *follow-through* (end of the swing). The required movement measurement accuracy during the swing execution is set to approximately 2 degrees [24]. We set the following requirements:

1. Static angle measurement error at address should be less than 2 degrees. The application measures the head position and inclination by detecting the gravity vector projection. The accelerometer error, which causes static angle error, should therefore not exceed 0.035 g_0_.
2. Dynamic angle measurement error during the swing should be less than 2 degrees. The gyroscope error, which causes rotation angle error, should therefore not exceed 0.67 deg/s if we want to stay under the 2 degree margin during the 3 s long swing execution time.

By comparing the above requirements to the figures in Table 2, we notice that the uncompensated accelerometer and gyroscope biases shown in Figure 2 are close to the application demands set above. Figure 2 shows that a certain number of measured smartphones could most likely be used in the developed biofeedback application without bias compensation. The rest of the smartphones must use at least occasional bias compensation to achieve the desired accuracy.

### 5.2. Application Configuration

The experimental biofeedback application implements the architecture and elements of the biofeedback system described in Section 2. We use smartphone's inertial sensors, the processing device (laptop) is nearby and under the control of an assistant, wireless headphones serve as the feedback device. With the appropriate attachment of the smartphone to the head of the golfer, we can achieve very good detection repeatability of different 3D head movements measured from the static start position in the swing setup phase. The application running on the laptop is designed in the LabVIEW™ development environment and is able to run on any compatible MS Windows operating system. The audio feedback signal is generated by the laptop and sent to the headphones on the golfer's head over the RF-ISM channel. In the current work, auditory feedback was chosen because auditory sensing is mostly unengaged during the golf swing exercise.

In the above configuration, the smartphone is used only as a sensing device. Practically all modern smartphones have enough processing power to handle the real-time signal processing of the above described biofeedback application. At the same time it can also perform the function of the feedback device (speakers or headphones output). Such compact version of the biofeedback system is much more attractive for a personal use and it is planned to be developed after proofing of the real-time biofeedback motor learning concept.

## 6. Results of the Application Tests

We conducted numerous measurements and experiments aimed primarily at testing the accuracy and precision of operation as well as the usability of the smartphone inertial sensors for the real-time biofeedback golf application. Because the accelerometer signals are used solely for head inclination at setup, we consider the gyroscope signals corresponding to head movements during the swing to be the most important.

Test measurements were recorded using an HD camera, and the recordings were used to help interpret the signals and derived results acquired from the inertial sensors. We focused primarily on the correct presentation of the sensor signals and the derived analysis results. Golf swings were performed by a professional golf player with a very consistent swing execution in terms of movement repeatability. The application analyzes swings from their takeaway phase to the end of the follow-through phase. The analysis was done on the swings that were subjectively characterized as successful by the professional golf player. The presentation of the smartphone sensor signals and the derived results is limited to the last 200 samples of each swing at the sampling frequency of 60 Hz, which yields a time window of 3.33 s. For the measurements, we used uncompensated smartphone inertial sensors (worst case).

**Figure 6:**
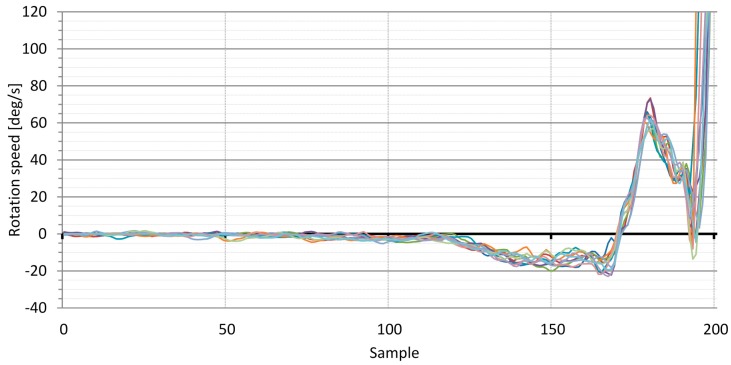
Series of swings performed by a professional golf player. Plots show gyroscope rotation speed around Z-axis in degrees per second. The presented last 200 samples of the swing, at sampling rate of 60 Hz, yield the analysis window of 3.33 s.

**Figure 7:**
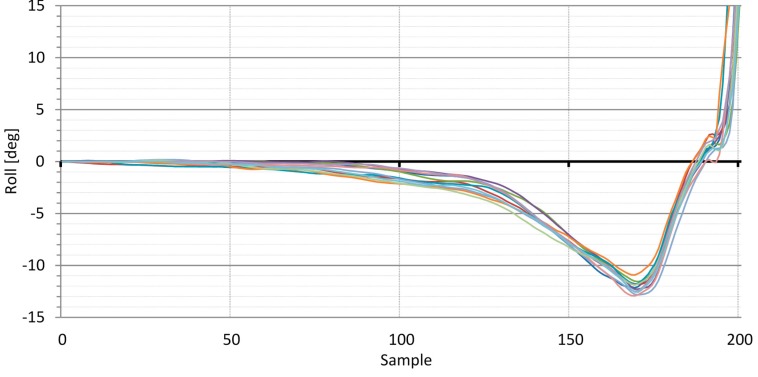
Series of swings performed by a professional golf player. The plots show the head roll in degrees for the last 3.33 s of each swing. The last 200 samples of the swing at a sampling rate of 60 Hz yields an analysis window of 3.33 s.

As an example, we present measurements of the gyroscope signal representing the rotation speed around the Z-axis (Figure 6) and the head roll of the player derived from this signal (Figure 7). As shown in Figure 6, the speed signals from the rotation of the player’s head indicate that the swing begins at approximately sample number 120. Before that point, the player’s head is at standstill, and gyroscope bias is responsible for most of the signal variation. The swing starts at approximately sample 115 (*takeaway*) when the player’s head starts to rotate according to the following swing execution pattern: (a) In the upswing, the player’s head rotates slowly to the left until the top of the backswing at approximately sample 170; (b) In the downswing, the player’s head rotates faster to the right until impact at approximately sample 190; (c) Following the body rotation in the follow-through phase of the swing, the player’s head rotates faster to the right. The results show that the swings of the professional golf player are very consistent.

Figure 7 shows that the plots up to approximately sample number 115 exhibit approximately linear characteristics. Upon close inspection of the HD video recordings, we confirmed that up to this point, the player’s head did not move to a visually perceivable extent. From the above observation, we can conclude that the approximate linear characteristics at the beginning of the plots are the sum of the angular drift caused by the gyroscope biases and of slight, visually imperceptible head movements of the player. Statistical parameters at the characteristic points of the swings presented in Figure 7 are as follow: (a) at the takeaway at sample 115, the standard deviation of the plots is 0.61 deg; (b) at the top of the backswing at sample 170, the standard deviation of the plots is 0.72 deg; and (c) at impact at sample 190, the standard deviation of plots is 0.84 deg.

The signal presented in Figure 6 (angular velocity in the Z-axis) and the derived results in Figure 7 (head roll) are only two of the many parameters made available by the developed application. The same explanations and conclusions can be drawn from other signals related to various head movements.

One important question arises: is the quality of the results acquired using uncompensated sensor signals high enough for implementation of the presented biofeedback idea? According to the second application requirement in Section 5.1, the dynamic angle measurement error should remain under 2 degrees for the duration of the golf swing analysis, meaning that the gyroscope bias should be less than 0.67 deg/s. As mentioned previously, some of the smartphones in Figure 2 satisfy this condition. The results presented in Figure 6 and Figure 7 were acquired using uncompensated smartphone gyroscopes. They are encouraging as they show that the measurement error, considering the slight player movement inconsistency, stays within 2 degrees.

Bias compensation reduces angular drift by an order of magnitude. For example, if the compensated bias error is 0.1 deg/s (see Figure 5), the 3 s analysis time frame yields an angular drift of 0.3 degrees. Measurement errors in this range are insignificant for the developed application. All of the perceivable signals from the compensated inertial sensors are caused by head movements.

The application test has answered the main question, and the developed application operates within the specified requirements even with uncompensated smartphone inertial sensors. For more advanced applications, longer analysis times, and more detailed movement analysis, bias compensation is necessary. The results from Section 3 show that even short averaging times (τ = 10 s) for bias compensation reduce the induced angular drift by a factor of 10. With more advance compensation techniques, we could achieve even better results (see options in Section 4.1).

## 7. Conclusions

The use of the inertial sensors in smartphones for motion capture and motion tracking in biomechanical biofeedback systems is limited due to their performance, which is expressed through the imprecision of their readings. These sensors are sufficiently precise for angular measurements in real-time biomechanical movement biofeedback applications if the time analysis window is not excessively large and if the movement dynamics fall within the sensor’s dynamic range and effective sampling frequency. Present day smartphone sensors are suitable for biofeedback applications with low to moderate movement dynamics containing accelerations in the range of ±8 g, angular velocities in the range of ±2000 deg/s, and requiring sampling frequencies in the range of 100 Hz [55].

We studied the smartphone sensor error sources and measured the accelerometer and gyroscope biases, identified the main causes for short-term and long-term bias variations, and quantified their precisions. The uncompensated accelerometer biases are in the range of ±40 m*g*_0_, and those of the uncompensated gyroscope are in the range of ±1 deg/s. These values restrict their use in biofeedback systems to short analysis windows up to a few seconds long. Bias compensation can significantly increase the range of sensor usability, both in prolonged analysis times and in applications with higher precision requirements. Compensation using averaging times between 10 s and 100 s primarily eliminates sensor white noise. The compensation reduces the bias by an order of magnitude, but the long-term bias variations caused by temperature changes may remain a problem.

We composed a table of predicted smartphone accelerometer and gyroscope analysis accuracies for motion capture in biomechanical biofeedback systems, see Table 2. The table includes various analysis time windows on which we base the presented bias compensation scenarios. These scenarios can be used as guidance for proper use of smartphone sensor signals in biofeedback applications.

We developed an experimental biofeedback application that showed the following: for loose biofeedback demands and short analysis times, the application operates within the specified requirements even with uncompensated inertial sensors; for stricter biofeedback demands, longer analysis times, and more detailed movement analysis, bias compensation is necessary.

The confirmation of the usability of smartphone inertial sensors for the experimental real-time biofeedback application is only the first step in a much larger project: implementation of biofeedback systems for motor learning. Some very modest experiments have already been conducted. These experiments indicate that the motor learning curve is much more favorable with the aid of biofeedback. We believe that such biofeedback systems are applicable to many similar examples in sport, fitness, healthcare, and other areas.

## Figures and Tables

**Table 1:** The main parameters of the MEMS accelerometer and gyroscope embedded in the iPhone 4.

Parameter	3-axis AccelerometerLIS331DLH	3-axis GyroscopeL3G4200D
Range	±2 g–±8 g	±250–±2000 deg/s
Sensitivity	1 ± 0.1 mg/dig	70 mdeg/s/dig
Bias error	±20 mg	±8 deg/s
Noise density	0.218 mg/sqrt(Hz)	0.03 deg/s/sqrt(Hz)
Data rate	0.5–1000 Hz	100/200/400/800 Hz

**Table 2:** Predicted analysis accuracy of accelerometer and gyroscope data in different bias compensation states and different analysis time frames. Red colored values indicate accuracies that are outside of the boundaries set for the example biofeedback application presented in the introduction section.

Sensor/Derived Parameter	Bias Error	Analysis Time Frame		
		1 s	3 s	10 s
Accelerometer *position error* [cm]	A Δ*a* = 40 mg_0_	**19.60**	**176.8**	**1964.0**
	B (*T_avg_* = 10 s), Δ*a* = 0.24 mg_0_ B (*T_avg_* = 100 s), Δ*a* = 0.08 mg_0_	0.10 0.04	1.0 0.3	**11.7** **3.7**
	C Δ*a* = 4 mg_0_ after one hour	2.00	**17.7**	**196.4**
Accelerometer *gravitation angle error* (static measurement) [deg]	A Δ*a* = 40 mg_0_	2.300		
	B Δ*a = 3*σ_A_(*T_avg_* = 10 s) = 0.24 mg_0_ B Δ*a = 3*σ_A_(*T_avg_* = 100 s) = 0.08 mg_0_	0.014 0.004		
	C Δ*a* = 4 mg_0_ after one hour	0.230		
Gyroscope *angle error* [deg]	A ΔΩ = 1.15 deg/s	1.15	**3.45**	**11.50**
	B ΔΩ = *3*σ_A_(*T_avg_* = 10 s) = 27 mdeg/s B ΔΩ = *3*σ_A_(*T_avg_* = 100 s) = 8.6 mdeg/s	0.03 0.01	0.08 0.03	0.27 0.09
	C ΔΩ = 86 mdeg/s after one h	0.09	0.26	0.86

**Table 3:** Predicted random walk errors induced by accelerometer and gyroscope white noise. Values in the table represent standard deviations.

Random Walk Error		Analysis Time Frame		
		1 s	3 s	10 s
Accelerometer VRW = 0.25 mg_0_	STD of the position error [cm]	0.14	0.74	4.48
	STD of the gravitation angle error [deg]	0.02		
Gyroscope ARW = 28 mdeg/s	STD of the angular error in [deg]	0.03	0.05	0.09
